# Recent Progress and Advances in HGF/MET-Targeted Therapeutic Agents for Cancer Treatment

**DOI:** 10.3390/biomedicines3010149

**Published:** 2015-03-19

**Authors:** Yilong Zhang, Rajul K. Jain, Min Zhu

**Affiliations:** 1 Department of Clinical Pharmacology, Modeling and Simulation, Amgen Inc., One Amgen Center Drive, Thousand Oaks, CA 91320, USA; E-Mail: minz@amgen.com; 2 Kite Pharma, Inc., 2225 Colorado Avenue, Santa Monica, CA 90404, USA; E-Mail: rjain@kitepharma.com

**Keywords:** HGF, MET, cancer treatment, new therapeutic agents, biomarker

## Abstract

The hepatocyte growth factor (HGF): MET axis is a ligand-mediated receptor tyrosine kinase pathway that is involved in multiple cellular functions, including proliferation, survival, motility, and morphogenesis. Aberrancy in the HGF/MET pathway has been reported in multiple tumor types and is associated with tumor stage and prognosis. Thus, targeting the HGF/MET pathway has become a potential therapeutic strategy in oncology development in the last two decades. A number of novel therapeutic agents—either as therapeutic proteins or small molecules that target the HGF/MET pathway—have been tested in patients with different tumor types in clinical studies. In this review, recent progress in HGF/MET pathway-targeted therapy for cancer treatment, the therapeutic potential of HGF/MET-targeted agents, and challenges in the development of such agents will be discussed.

## 1. Introduction

Hepatocyte growth factor (HGF) and its receptor MET regulate multiple normal cellular functions, including proliferation, survival, motility, and morphogenesis. HGF/MET signaling appears to be a critical pathway in a variety of malignancies, and thus it has become a potential target for cancer therapeutics [1].

One of the key considerations in developing anticancer drugs is their selectivity against cancer cells while sparing normal cells. Among a group of biologically important targets for cancer intervention, MET is a receptor tyrosine kinase (RTK) that has low activity in normal tissues but is dysregulated in many tumor types [2]. HGF, also known as scatter factor, is the only known ligand to the MET receptor. HGF and MET are both required for normal mammalian development and are widely expressed in a variety of tissues, while their expression level is normally very low. The HGF/MET pathway is involved in multiple cellular responses (e.g., proliferation, survival, differentiation, motility, and invasion) as well as physiological responses (e.g., tissue repair and regeneration).

Evidence directly linking MET to cancer has come from the original discovery of missense *MET* mutations in patients with hereditary papillary renal cell carcinomas [3]. Somatic *MET* mutations in many other cancers continue to be discovered, such as in gastric, head and neck, liver, ovarian, non-small cell lung, and thyroid cancers [4,5,6,7,8,9,10,11,12,13]. MET is commonly activated by elevated HGF levels or somatic mutations in the HGF promoter region that result in increased HGF levels [14]. In tumor biopsies, MET was found to be overexpressed in selected types of solid tumors, and HGF was widely detected in the intratumoral spaces of solid tumors [15]. Collective biochemical and genetic evidence suggests an association between dysregulated HGF/MET signaling and selected human cancers.

Research on anticancer drugs targeting HGF/MET signaling in solid tumors has been under preclinical and clinical investigation for the past two decades [16,17,18,19,20,21,22]. A number of novel HGF/MET-targeting agents, either as therapeutic proteins or as small molecules, has been tested in patients with cancer, and some of them showed encouraging results in clinical studies (Table 1).

**Table 1:** HGF/MET inhibitors in clinical development.

Compound	Modality	Target(s)	Company	Cancer Type	Development Phase
Rilotumumab (AMG 102)	Antibody	HGF	Amgen	Gastric, lung, colon, brain, ovary, renal	2/3
Ficlatuzumab (AV-299)	Antibody	HGF	AVEO Pharmaceuticals	Lung	1/2
HuL2G7 (TAK701)	Antibody	HGF	Galaxy Biotech	Solid tumors	1
Onartuzumab (MetMab)	Antibody	MET	Genentech/Roche	Lung, colon, breast	2/3
AMG 337	Small molecule	MET	Amgen	Solid tumors	1/2
INC 280	Small molecule	MET	Novartis/Incyte	Renal, brain, liver, lung, melanoma, head and neck	2
Tivantinib (ARQ 197)	Small molecule	MET	ArQule/Daiichi–Sankyo/Kyowa Hakko Kirin	Lung, colon, breast, liver, prostate, myeloma	2/3
Crizotinib (PF-2341066)	Small molecule	MET, ALK	Pfizer	Lung, lymphoma	2/3
Cabozantinib (XL 184)	Small molecule	MET, VEGFR2, RET, KIT, AXL, FLT3	Exelixis/Bristol–Myers Squibb	Lung	2/3
Foretinib (XL 880)	Small molecule	MET, VEGFR2, PDGFR, RON, FLT-2, FLT-3, AXL, TIE2	Exelixis/GlaxoSmithKline	Lung, breast, liver, renal, stomach, head and neck	1/2	
Golvatinib (E7050)	Small molecule	MET, VEGFR2	Eisai Inc.	Liver, head and neck, stomach	1	
MGCD265	Small molecule	MET, VEGFR2, RON, TIE2	MethylGene	Lung	1/2	
BMS-777607	Small molecule	MET, RON	Bristol-Myers Squibb	Solid tumors	1/2	

Clinical development information of HGF/MET inhibitors is from [23]. HGF: hepatocyte growth factor; ALK: anaplastic lymphoma kinase; FLT: FMS-like tyrosine kinase-3; PDGFR: platelet-derived growth factor receptor; VEGFR: vascular endothelial growth factor receptor.

In this review article, we will discuss the recent progress and advances in HGF/MET pathway-targeted therapy for cancer treatment, the therapeutic potential of HGF/MET-targeted agents, and challenges in the development of such agents, focusing on the following aspects:

(1) Mechanism of action: targeting the ligand (*i.e.*, HGF) *vs.* targeting the receptor (*i.e.*, MET)
(2) Modality of therapeutic agents: small molecules *vs.* therapeutic proteins
(3) Treatment strategies: monotherapy and combination therapy
(4) Value and clinical implications of biomarkers for the HGF/MET pathway
(5) Challenges and perspectives on the development of HGF/MET therapeutics

## 2. Mechanism of Action: Targeting the HGF Ligand *vs.* Targeting the MET Receptor

**Figure 1:**
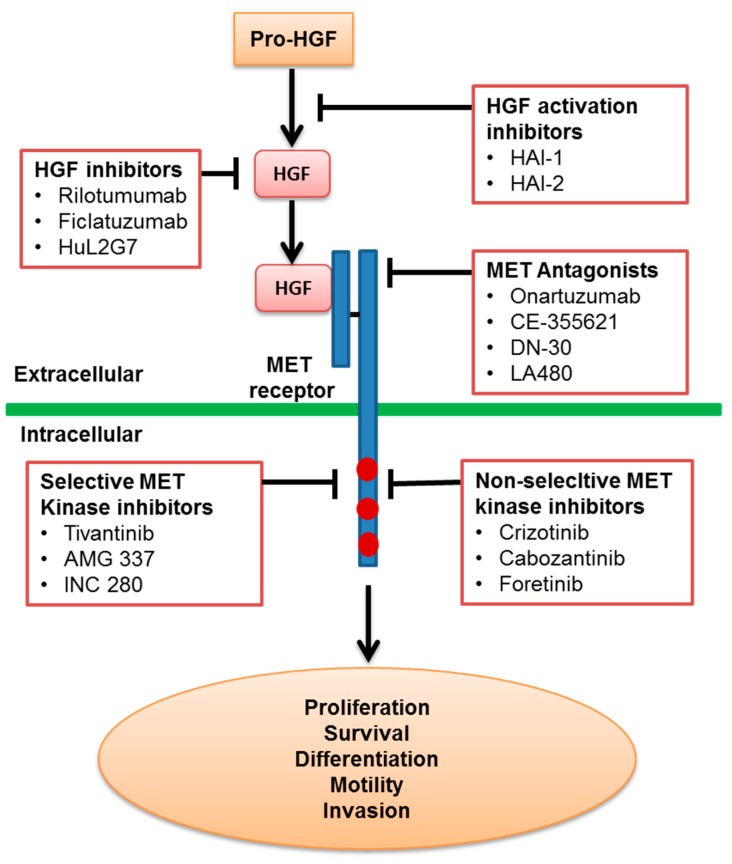
Representative examples of different mechanism classes of HGF and MET inhibitors. HGF: hepatocyte growth factor.

Based on their mechanisms of action, the HGF/MET-targeting agents can be generally categorized into agents that either target the HGF ligand or those that target the MET receptor. For agents targeting the ligand, they can be further categorized into either HGF activation inhibitors that prevent the cleavage of pro-HGF into the active form of HGF or HGF inhibitors that block the direct binding of HGF to the MET receptor. For agents targeting the MET receptor, they can further be categorized into either MET antagonists that bind the receptor without activation of downstream signaling or MET tyrosine kinase inhibitors (TKIs) that target the adenosine triphosphate (ATP)-binding site of the receptor and block receptor transphosphorylation (Figure 1).

### 2.1. Inhibition of HGF Activation

The activation of HGF from its inactive precursor pro-HGF is a critical step in HGF functioning [24]. This process is governed by a number of proteases, including HGF activator (HGFA), matriptase, hepsin, and urokinase-type plasminogen activator (uPA), all of which have the ability to convert inactive pro-HGF to active HGF. Two recently identified HGF activation inhibitors (HAIs), HAI-1 and HAI-2, have been shown to block HGF activation. Thus, the degree of bioactive HGF formation is regulated by the activity between these activators and inhibitors [25,26,27,28]. The elevation of HGFA and reduction of HAIs have been reported in a variety of tumors, indicating that the disruption of the balance between HGF activation and inhibition may favor tumor formation [29,30,31]. In addition, clinical data show that HAIs are inversely correlated with patient prognosis in different types of cancers, which further strengthens the potential role of HAIs as novel prognostic markers [32,33,34,35,36,37]. Recent research evidence suggested the therapeutic potential of recombinant HAI proteins for the treatment of a variety of cancers, including prostate, breast, and ovarian cancer [35,38,39]. However, the development of HAIs in cancer treatment is still in the early stages. Further work is required to determine the true clinical value of HAIs as cancer therapeutic agents or as prognostic biomarkers.

### 2.2. Inhibition of HGF Binding to the MET Receptor

HGF inhibitors bind and neutralize HGF and block HGF from binding to the MET receptor, thus preventing the downstream activation of the pathway. To date, only a few monoclonal antibodies (mAbs) that directly bind against HGF, including ficlatuzumab, HuL2G7, and rilotumumab, have been studied in clinical trials.

Ficlatuzumab (AV-299) is a humanized anti-HGF antibody currently in phase 2 development. Clinical data from phase 1 trials have shown that ficlatuzumab was well tolerated as a monotherapy and in combination with the epidermal growth factor receptor (EGFR) inhibitors erlotinib and gefitinib [40,41,42]. Preliminary clinical activity (*i.e.*, partial response) was observed in patients with non-small cell lung cancer (NSCLC) from these early trials [42]. Although a phase 2 trial in treatment-naïve Asian patients with NSCLC did not show statistically significant differences in overall response rate (ORR) and progression-free survival (PFS) by adding ficlatuzumab to gefitinib in the intention-to-treat (ITT) population, the combination of ficlatuzumab and gefitinib demonstrated a trend toward ORR and PFS improvement in a subset of patients with both EGFR-sensitizing mutations and low MET biomarker levels. In addition, preliminary overall survival (OS) results suggested that the addition of ficlatuzumab to gefitinib may significantly prolong survival in patients with high stromal HGF (*p* = 0.03) [43]. These observations warrant further evaluation of ficlatuzumab in NSCLC.

Rilotumumab (AMG 102) is the first HGF ligand inhibitor to reach phase 3 development. Its antitumor effects have been evaluated in the last decade as a monotherapy or in combination with other anticancer therapies in a variety of tumor types [44,45,46,47,48,49,50]. In a phase 2 trial in patients with advanced gastric cancer, rilotumumab in combination with epirubicin, cisplatin, and capecitabine (ECX) suggested potentially improved efficacy outcomes (PFS and OS) compared to ECX alone [51]. Data from this study support the evaluation of rilotumumab in patients with advanced gastric cancer and positive MET expression. However, the preliminary results from a phase 3 study did not confirm the phase 2 findings. Thus, the development of rilotumumab in gastric cancer was stopped.

HuL2G7 (TAK-701) was one of the earliest mAbs developed that effectively neutralizes HGF. There was one phase 1 study conducted in advanced nonhematologic malignancies, in which HuL2G7 displayed a favorable safety profile with some indication of activity [52].

### 2.3. MET Antagonists

Activation of the HGF/MET pathway requires the dimerization of the MET receptor upon the binding of the active form of HGF, which leads to kinase autoactivation. MET antagonists compete with HGF for MET binding, resulting in the degradation of MET and subsequent inactivation. Several MET antagonists have been discovered, including onartuzumab, CE-355621, DN-30, and LA480 [21,53,54,55]. While therapeutic strategies which target MET should in principle be similarly efficacious to those targeting HGF, MET antagonists have been difficult to develop as many of them are bivalent (two-arm) antibodies that induce crosslinking and subsequent downstream signal activation, thereby mimicking HGF.

Onartuzumab is the only monovalent MET antibody that has been tested in clinical studies to date. Phase 1 data had shown that onartuzumab was well tolerated when administered alone or in combination with bevacizumab in patients with cancer [56]. A phase 2 clinical study using onartuzumab in combination with erlotinib for advanced NSCLC showed significant clinical improvement on PFS and OS in MET-positive NSCLC [57,58]. However, the results from phase 2 were not replicated in a larger phase 3 trial, and the study was stopped due to a lack of clinically meaningful efficacy. At this time, further development of onartuzumab has been halted.

### 2.4. MET Kinase Inhibitors

Another approach for inhibiting the MET pathway is through MET kinase inhibitors, which target MET intracellularly. Several small-molecule MET kinase inhibitors have entered clinical development over the past decade, including selective MET kinase inhibitors (e.g., tivantinib (ARQ 197), AMG 337, and INC 280) and nonselective MET kinase inhibitors (e.g., crizotinib (PF02341066), cabozantinib (XL 184), and foretinib).

Tivantinib (ARQ 197) is the first non-ATP-competitive agent that selectively targets the MET kinase. In clinical trials, treatment with tivantinib has been generally well tolerated and has shown some clinical activity in patients with cancer [59,60,61,62,63,64,65,66,67,68]. In a large phase 3 trial of tivantinib plus erlotinib for the treatment of patients with advanced or metastatic NSCLC, although not statistically significant, OS favored the treatment arm of tivantinib plus erlotinib compared to the erlotinib alone arm (median OS of 12.9 months *vs.* 11.2 months, hazard ratio (HR) = 0.89, *p* = 0.4). Numerical trends toward improved PFS and ORR were also seen favoring the treatment arm [69]. In a phase 2 trial of tivantinib as a single agent for the treatment of hepatocellular carcinoma (HCC), significant improvement in OS (7.2 months *vs.* 3.8 months, HR = 0.38, *p* = 0.01) was observed in patients with high tumor MET expression [65]. Two follow-up phase 3 trials in patients with HCC have been initiated.

AMG 337 is a selective MET kinase inhibitor that is currently in phase 2 clinical development. Preliminary results showed that AMG 337 has potential as a therapeutic agent for treating *MET*-amplified solid tumors [70]. This inhibitor is currently being evaluated in patients with *MET*-amplified gastric/esophageal adenocarcinoma and other solid tumors.

INC 280 is another highly selective, oral small-molecule MET inhibitor. In a phase 1 study, it was well tolerated in patients with advanced MET-dependent solid tumors [71]. Dose expansion in the phase 1 study in NSCLC, HCC, and other tumors as well as phase 2 studies in various solid tumors are ongoing.

Other types of MET inhibitors in clinical development are nonselective, multikinase inhibitors. For example, crizotinib targets MET and ALK [72]; cabozantinib targets MET, VEGF2, AXL, RET, KIT, and FLT3 [73]; and foretinib targets MET, VEGFR2, PDGFR, RON, FLT-1, FLT-4, and TIE2 [74].

Crizotinib earned accelerated approval by the United States Food and Drug Administration (US FDA) in 2011 for the treatment of ALK-positive NSCLC. Subsequently, the drug was granted regulatory approval in 2013 based on its superiority on PFS and ORR compared with chemotherapy [75]. Recent analysis of previously unreported results has shown that crizotinib produced either disease stabilization or tumor response in patients with NSCLC and high *MET* amplification, suggesting that crizotinib may be a potential agent for the treatment of *MET*-amplified NSCLC [76].

Cabozantinib strongly inhibited the self-renewal potential and *in vivo* growth of a MET-positive tumorigenic subpopulation of giant cell tumor of bone stromal cells, and thus it may be an effective therapeutic option for the targeted elimination of a tumorigenic stromal subpopulation in patients with nonresectable or recurrent giant cell tumor of bone [77]. Cabozantinib was granted marketing approval by the US FDA for treating patients with medullary thyroid cancer based on significant PFS prolongation (11.2 *vs.* 4.0 months, HR = 0.28, *p* < 0.0001) and ORR improvement (28% *vs.* 0%) [78] and is currently undergoing multiple phase 3 trials in a variety of indications, including castration-resistant prostate cancer, metastatic renal cell carcinoma, and HCC.

Similar to cabozantinib, foretinib, golvatinib, and MGCD265 are dual MET/VEGF2 inhibitors. In addition, foretinib also inhibits PDGFR, RON, FLT-2, FLT-3, AXL, and TIE2 with low potency. BMS-777607 is a dual inhibitor of MET and RON. Early-phase clinical data for these multikinase inhibitors have demonstrated favorable safety profiles either as a single agent or in combination with chemotherapy [79,80]. Clinical evaluations on the therapeutic benefit of these agents are currently ongoing.

## 3. Modality of Therapeutic Agents: Clinical Features of Small Molecules and Therapeutic mAbs

Based on the modality of therapeutic agents, HGF/MET-targeting agents can be classified as either therapeutic mAbs or small molecules. Both mAbs and small-molecule HGF/MET inhibitors have shown clinical benefits in patients with cancer, and they are different in terms of pharmacological properties and their underlying mechanisms of action.

mAbs are large proteins, and are difficult to penetrate cell membrane; they mainly target HGF in the extracellular circulation system or target the MET receptor on the cell surface. While small molecules have the advantage of crossing the cell membrane to interact with targets inside a cell, they are usually designed to interfere with the enzymatic activity of the MET tyrosine kinase. The mAbs are highly specific for their target, while the TKIs vary from being highly specific class I molecules to class II molecules that have multikinase activity and are less potent against MET.

Interestingly, for HGF/MET inhibitors that are currently under clinical evaluation, all agents targeting circulating HGF or the extracellular MET receptor are mAbs, while all agents targeting the MET tyrosine kinase are small molecules. However, there appears to be no clear difference in the spectrum of indications between mAbs and small molecules. The major target indications for HGF/MET inhibitors are solid tumors, including gastric cancer, NSCLC, HCC, and thyroid cancer (Table 1).

Although mAbs can only act on extracellular targets, they are generally more specific for the targets than small-molecule inhibitors and thus are expected to have better safety profiles than small molecules. This has been demonstrated by rilotumumab, ficlatuzumab, HuL2G7, and onartuzumab as these molecules were well tolerated in patients with cancer up to the highest doses administered (either 20 mg/kg once every 2 weeks (q2w) or 30 mg/kg once every 3 weeks (q3w)). There were no dose-limiting toxicities (DLTs) reported for any of these mAbs (Table 2). The most commonly observed toxicities were fatigue, nausea, and peripheral edema at grade 1 or grade 2. The immunogenicity of these HGF/MET mAbs appeared to be limited [41,44,52,56].

In contrast, the adverse effects associated with small-molecule inhibitors appeared to be compound-specific, and DLTs have been observed with small-molecule MET kinase inhibitors [59,70,71,79,80,81,82,83]. Nevertheless, as a class of agents, they appear to have generally acceptable toxicity profiles. Of the TKIs, in general, the more specific class I agents may have more favorable side effect profiles than the class II agents, although class I and class II agents have not been directly compared. Current data have indicated that peripheral edema could emerge as a class effect, as it has been observed with both MET-targeting [57] and HGF-targeting agents [51].

Multiple clinical trials, ranging from phase 1 to phase 3, of HGF/MET axis-targeting agents are ongoing or have been completed. Some studies have generated negative results, whereas others have generated intriguing signals of potential efficacy. To date, no agent has generated conclusive data in a large enough sample set to garner a marketing indication from a global health authority. However, few observations have begun to emerge.

With respect to efficacy, a target-specific agent might not have therapeutic advantage over a dual- or multi-target agent, since most tumors are more frequently linked to defects of more than one target. To date, the only US FDA-approved MET pathway-related agents, crizotinib and cabozantinib, are dual- or multi-tyrosine kinase inhibitors, indicating that blocking MET jointly with other cancer-related pathways might be more beneficial for cancer treatment.

**Table 2:** Summary of pharmacokinetic parameters and top FIVE grade ≥ 3 adverse events (AEs) of selected HGF/MET inhibitors when administered as a single agent.

Compound	Top Five Grade ≥ 3 AE (%)	MTD or the Highest Tested Dose	CL	*V*_d_	*t*_1/2_
Rilotumumab (AMG 102) [44]	Hypoxia (3)	20 mg/kg q2w	0.104–0.176 mL/h/kg	59 mL/kg [84]	14.5–22.0 days
	Dyspnea (3)				
	Upper gastrointestinal hemorrhage (3)				
	Colonic fistula (3) *				
Ficlatuzumab (AV-299) [41]	Hypokalemia (17)	20 mg/kg q2w	0.16 ± 0.06 mL/h/kg	49.4 ± 11.0 mL/kg	225 ± 74 h
	Peripheral edema (8)				
	Fatigue (4)				
	Diarrhea (4)				
	Vomiting (4)				
HuL2G7 (TAK701) [52,85]	Gastrointestinal ileus (2)	20 mg/kg q2w	NR	NR	8.6–14.1 days
	Pleural effusion (2)				
	Urinary tract infection (2)				
	Dyspnea (1) *				
Onartuzumab (MetMab) [56]	Edema peripheral (9)	15 mg/kg q3w	6.85 ± 1.94 mL/day/kg	96.8 ± 23.4 mL/kg	11.5 ± 5.54 days
	Abdominal pain (3)				
	AST increased (3)				
	Pyrexia (3)				
	Hyponatremia (3)				
AMG 337 [70]	Headache (9)	300 mg QD	NR	NR	4.6–6.9 h
	Fatigue (5)				
	Dyspnea (5)				
	Vomiting (3)				
	Nausea (1)				
INC 280 [71]	Blood bilirubin increased (12)	600 mg BID	NR	NR	2.3–6.4 h
	Anemia (9)				
	Fatigue (9)				
	Hyponatremia (9)				
	Hypophagia (6)				
Tivantinib (ARQ 197) [59]	Anemia (4)	360 mg BID	NR	NR	1.5–2.7 h
	Neutropenia (3)				
	Leukopenia (1)				
	Thrombocytopenia (1)				
	Nausea (1)				
Crizotinib (PF-2341066) [75,86]	ALT elevation (17)	250 mg BID	100 L/h at single dose; 60 L/h at steady state	1772 L	42 h
	Neutropenia (12)				
	AST elevation (9)				
	Lymphopenia (9)				
	Hypophosphatemia (5)				
Cabozantinib (XL 184) [81]	Fatigue (10)	175 mg QD	4.4 L/h	349 L	91.3 ± 33.3 h
	Palmar plantar erythrodysesthesia (10)				
	Increased lipase (10)				
	Diarrhea (7)				
	Decreased weight (6)				
Foretinib (EXEL 2880) [82]	Hypertension (24.3)	80 mg QD	83 L/h	NR	NR
	Fatigue (18.9)				
	Nausea (10.8)				
	Diarrhea (10.8)				
	Dehydration (10.8)				
Golvatinib (E7050) [87]	Fatigue (14.7)	400 mg QD	NR	325–707 L	NR
	Decreased appetite (8.8)				
	Renal impairment (2.9)				
	Elevated GGT (2.9)				
	Elevated ALP (2.9)				

* Only four AEs grade ≥ 3 were reported. HGF: hepatocyte growth factor; MTD: maximum tolerated dose; NR: not reported; q2w: once every 2 weeks; q3w: once every 3 weeks; QD: once daily; BID: twice daily; CL: clearance; *V*_d_: volume of distribution; *t*_1/2_: half-life.

For any agent to succeed in this space, proper patient selection is of paramount importance [88]. Various selection schemes leverage *MET* protein level, *MET* gene amplification, and MET mutational status. Within each selection strategy are various cutoffs, reagents, and methodologies to define an overall biomarker “positive” or “negative” status. As an example, in a review of the high-profile study of onartuzumab in NSCLC that was recently reported to be negative [89], it has been speculated that patient selection may have contributed to the negative study result [88,90], although this observation is not universally accepted [58].

Furthermore, mAbs and small-molecule HGF/MET inhibitors differ in other pharmacological properties (Table 2). Rilotumumab, ficlatuzumab, and onartuzumab are large proteins that can only be administered intravenously, whereas tivantinib, AMG 337, PF04217903, and other small-molecule MET kinase inhibitors are generally administered orally.

Unique pharmacokinetic characteristics were observed for mAbs and small-molecule HGF/MET inhibitors (Table 2). Due to the difference in molecular weight (150 kDa for mAbs *vs.* ~500 Da for small molecules), mAbs have limited tissue distribution and usually have a similar volume of distribution (*V*_d_) as plasma volume. In contrast, small molecules have different chemical- and protein-binding properties and can extensively distribute into tissues and have a much larger volume of distribution than mAbs (Table 2). Thus, theoretically small molecules seem to have a therapeutic advantage for treatment of solid tumors possibly because they can be delivered into tumors relatively more easily than mAbs. Nevertheless, more and more mAbs have been approved for the treatment of solid tumors, among them are some mAbs that target circulating ligands (e.g., Avastin^®^) which are independent of tissue penetration, while others target receptors (e.g., Herceptin^®^), which may rely on their specific ways to reach target tissues to exert pharmacological activities. Furthermore, mAbs normally have much longer circulating time in the blood than small molecules, which compensates for the slow process of drug distribution.

The half-lives of mAbs are about 9–18 days, which renders a q2w or q3w dosing regimen possible. Generally, the vast majority of mAbs is eliminated by catabolism [91]. Collective evidence supports an important role for FcRn (the Fc-receptor of the neonate) in immunoglobulin G (IgG) catabolism by forming FcRn-bound IgG complexes that protect IgG from degradation. After IgG molecules are taken into the endosomes of catabolic cells by pinocytosis, H^+^ enters the endosomes, resulting in increased IgG binding to FcRn at a pH lower than the physiological value. FcRn-bound IgG is returned to the central compartment, and unbound IgG proceeds to the lysosome and undergoes proteolytic degradation. Therefore, IgG elimination is likely dominated by affinity for the FcRn receptor.

In contrast, the half-lives of small-molecule MET inhibitors were in a range of hours, requiring once daily (QD) or twice daily (BID) dosing regimens. The small-molecule MET inhibitors (*i.e.*, crizotinib, cabozantinib and foretinib) are predominantly metabolized by cytochrome P450 (CYP) enzymes [92,93,94]. Some of them (*i.e.*, crizotinib) could be CYP450 inhibitors or inducers [92]. Therefore, co-administration of these small-molecule MET inhibitors with chemotherapeutic agents or other small-molecule concomitant medications may be restricted due to potential drug–drug interactions. In contrast, mAbs have advantages for drug combinations considering clinically meaningful interactions between mAbs and small-molecule agents are not likely to occur [91,95].

## 4. Treatment Strategies: Monotherapy and Drug Combination

The era of targeted therapy has the promise to deliver highly precise therapeutics with minimal off-target toxicity and significantly improved safety profiles compared with traditional cytotoxic drugs. While some targeted therapies have significant efficacy as monotherapy, such as imatinib for chronic myelogenous leukemia, others have limited single-agent activity but yield synergistic effects when used in drug combinations.

A prevalent hypothesis is that a targeted agent is more likely to produce single-agent activity when the targeted abnormality is the key driver aberration, while combination approaches are required when multiple pathways need to be inhibited. With HGF/MET-targeting agents, both monotherapy and combination approaches have been and are continuously being explored.

### 4.1. Monotherapy

One hypothesis in the field is that *MET* gene amplification is more likely to represent a driver abnormality and hence be more susceptible to single-agent approaches, compared to a scenario where tumor cells express MET on the cell surface in the absence of gene amplification. Additionally, it is speculated that with *MET* amplification, there may be sufficient protein expression level to induce receptor autophosphorylation and thereby achieve a “ligand-independent” signaling state. While there have been reports of single-agent activity in *MET*-amplified settings [70,76], limited single-agent activity has been observed with agents targeting the HGF/MET axis upstream of the MET kinase domain [44,56].

The other case of monotherapy is one molecule targeting multiple pathways, as seen with crizotinib, cabozantinib, foretinib, golvatinib, and MGCD265. To date, HGF/MET inhibitors have been evaluated in patients with various cancer types in clinical trials, nearly half of which focused on efficacy evaluation. Monotherapy was tested in about 40% of the efficacy trials with HGF/MET inhibitors [96]; some of them showed antitumor activities. Nevertheless, most of the HGF/MET inhibitors showed promising results against multiple targets when used in drug combinations, likely due to the complexity of tumor biology [51,57,68].

### 4.2. Combination of Chemotherapy with HGF/MET Inhibitors

Most tumors are more frequently linked to defects of more than one target. As a result, targeting a single pathway may be insufficient to fully control tumor growth. These findings seem consistent with those of other targeted therapies in cancer treatment [78,79].

Combination of targeted therapy with chemotherapy is not new in oncology. Several HGF/MET inhibitors, especially HGF/MET mAbs, have been evaluated in combination with different chemotherapies in various oncology indications. This has been exemplified with rilotumumab in combination with ECX for the treatment of gastric cancer, and onartuzumab in combination with paclitaxel and cisplatin (or carboplatin) for the treatment of NSCLC [51,85]. Although some clinical outcomes support the combination of HGF/MET inhibitors and chemotherapies as a potential effective approach in cancer treatment, the underlying mechanism of their combination effects is still unclear.

### 4.3. Combination of Targeted Therapy with HGF/MET Inhibitors

Another combination approach being evaluated in patients with cancer is MET inhibitors plus other targeted therapies, including VEGF, EGFR, and ALK.

Recent data from a series of experiments in animal models suggested that inhibition of MET and VEGF simultaneously not only reduced tumor size but also reduced invasion and metastasis, thereby improving the treatment effect with either VEGF or MET inhibitors alone [97,98]. The promising clinical results of cabozantinib, a dual inhibitor of both MET and VEGF, seem to strongly support this clinical strategy [81]. In a phase 3 medullary thyroid cancer clinical trial, cabozantinib showed an almost three-fold improvement in median PFS (11.2 months) compared to placebo (4 months) [78]. Clinical trials to evaluate other MET/VEGF dual inhibitors (golvatinib, MGCD265) or the combination effect of MET inhibitor crizotinib with VEGF inhibitors (sunitinib and axitinib) are ongoing.

Furthermore, for the treatment of HCC, the HCC samples with high levels of phosphorylated MET were found to be resistant to adjuvant sorafenib treatment. The dual blockade of MET and VEGFR2 by cabozantinib has significant antitumor activities in HCC, and the activation of MET in HCC may be a promising efficacy-predicting biomarker [99].

**Figure 2:**
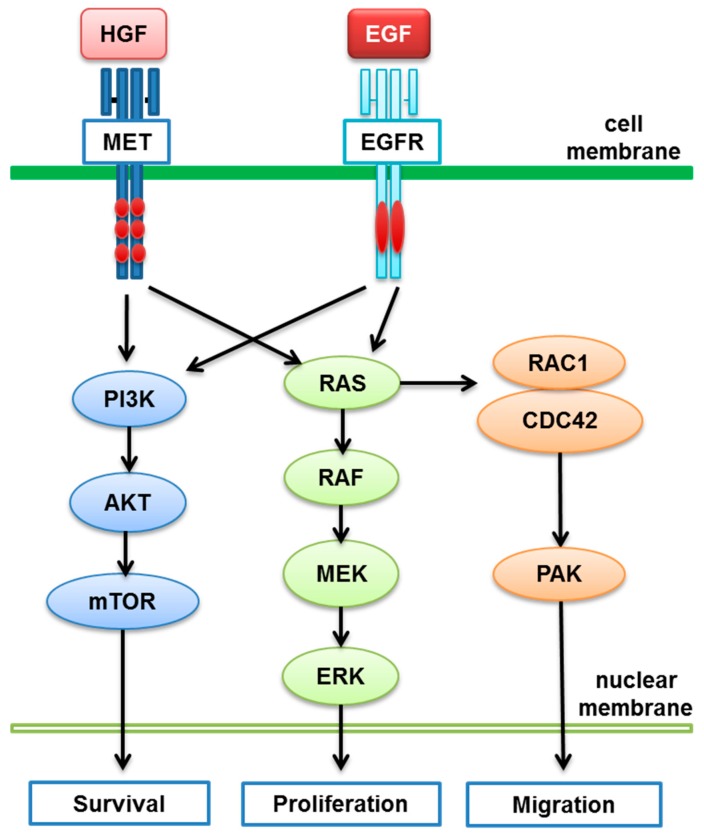
Crosstalk between the MET and EGFR pathways. HGF: hepatocyte growth factor; EGF: epidermal growth factor.

Research in the last decade has revealed that crosstalk between the MET and EGFR pathways on cell’s survival, proliferation and migration (Figure 2) may play a key role in enhancing cancer cell development and progression in NSCLC. Preclinical studies have shown the synergistic effect between HGF and epidermal growth factor (EGF) on cancer cell proliferation and downstream activation of signal transduction, and the potential application of both MET and EGFR TKIs in a synergistic fashion in cancer treatment [100,101]. Several clinical trials were conducted to determine the combination effect of MET inhibitors and EGFR inhibitors in NSCLC. Attractive results with MET antibodies in combination with an EGFR inhibitor (erlotinib) have been reported in phase 2 trials for onartuzumab and tivantinib [57,68]; however, the results were not confirmed in phase 3 trials. Other trials to evaluate the combination effect of erlotinib and MET inhibitors, (e.g., MGCD265 and cabozantinib in NSCLC) are currently in progress.

Another potential target combination is MET/ALK inhibition. Recent phase 3 results have shown striking activity of the dual MET/ALK inhibitor crizotinib in patients with NSCLC carrying an *EML4-ALK* fusion [102]. Although the therapeutic effect in this patient population may be mainly due to the inhibition of ALK, the drug effect on MET inhibition could also contribute to the overall treatment benefit. In addition, foretinib, a multitarget inhibitor of MET, VEGF2, RON, and AXL, has shown tumor reduction in patients with breast cancer resistant to inhibitors of EGFR (such as erlotinib) or EGFR and ERBB2 (such as lapatinib) [94].

### 4.4. Drug Combination against Drug Resistance

Drug combinations have been continuously evaluated for the reduction of drug resistance. It was found in a phase 1 trial evaluating the combination of the selective MET inhibitor tivantinib plus erlotinib that amplification of the *MET* gene can promote tumor resistance to EGFR inhibition, suggesting drug resistance to EGFR inhibition can be mediated through parallel activation of downstream signal transducers. From a mechanism point of view, *MET* amplification may contribute to resistance to EGFR inhibition through increased MET heterodimerization with human EGFR-related protein 3 (HER3) and activation of the PI3K/AKT cell survival pathway. Thus, dual EGFR–MET inhibition may overcome this resistance [67]. Preclinical experiments (cell-based tests or xenograft models) have demonstrated that dual EGFR–MET inhibition can overcome resistance to EGFR tyrosine kinase inhibition.

Furthermore, the MET/HGF axis plays a major role in the development of chemoresistance so that HGF/MET inhibitors may enhance sensitivity of cancer cells to the chemotherapy. For instance, ovarian cancer is one of the most aggressive female reproductive tract tumors. Paclitaxel is widely used for treatment but chemotherapeutic resistance is often acquired. It was found that miR-31 was downregulated in KFr13Tx cells and re-introduction of miR-31 resensitized them to paclitaxel both *in vitro* and *in vivo*. The decrease in MET was found to be correlated with higher sensitivity to paclitaxel and cotreatment of KFr13Tx cells with MET inhibitors sensitized the tumor cells to paclitaxel. The study demonstrated miR-31-dependent regulation of MET for chemoresistance of ovarian cancer, raising the possibility that combination therapy with a MET inhibitor and paclitaxel will increase paclitaxel efficacy [103].

HER kinase activation confers resistance to MET tyrosine kinase inhibition in MET oncogene-addicted gastric cancer cells. It was hypothesized that collateral growth factor receptor pathway activation can overcome the effects of MET inhibition in *MET*-amplified cancer cells by reactivating key survival pathways. Experiments were conducted to treat *MET*-amplified GTL-16 and MKN-45 gastric cancer cells with the highly selective MET inhibitor PHA-665752, and it was found to abrogate MAPK and PI3K/AKT signaling. The results illustrate that highly targeted MET tyrosine kinase inhibition leaves MET oncogene-addicted cancer cells vulnerable to HER kinase-mediated reactivation of the MEK/MAPK and PI3K/AKT pathways, providing a rationale for combined inhibition of MET and HER kinase signaling in *MET*-amplified tumors that coexpress EGFR and/or HER-3 [104].

Patients with advanced stages of NSCLC were frequently detected with HGF upregulation. *In vitro*, HGF reduced expression level of apoptosis-inducing factor (AIF) and cisplatin sensitivity in NSCLC cells. The effect of HGF was via the MET receptor and the downstream effector, focal adhesion kinase (FAK). It was found that expression level of AIF inversely correlated with the expression of positive NSCLC markers, such as dihydrodiol dehydrogenase (DDH), MET, short oncostatin M receptor (OSMR), matrix metalloproteinase (MMP)-1, and HER2/neu, which were closely associated with drug resistance, tumor recurrence, metastasis, and poor prognosis. Addition of HGF inhibited AIF expression level in HER2/neu-silenced cells, indicating that both HGF and HER2/neu affect drug resistance by regulating AIF expression level in NSCLC [105].

In summary, the choice of monotherapy or drug combination therapies is dependent on drug mechanisms of action, target indications, and mechanisms of drug synergy or resistance.

## 5. Value and Clinical Implications of HGF/MET Biomarkers

MET signaling has been shown to mediate a variety of cellular processes related to invasive growth. Under normal physiological conditions, these signals are crucial for mediating related biological processes, such as tissue patterning during embryogenesis, organ homeostasis, and wound repair. Since tumorigenesis may involve the dysregulation of the MET signaling pathway, changes in HGF or MET signaling are expected to be potential biomarkers for understanding cancer development and the effect of drugs.

### 5.1. HGF/MET Biomarkers in Cancer Development

Most biomarkers studied to date were related to its functions in cancer development. The commonly measured biomarkers are circulating HGF and MET levels, MET protein expression levels in tumor tissues, and *MET* gene amplification, copy number, and mutation, as well as markers related to the crosstalk of related pathways.

#### 5.1.1. Circulating HGF

An increase in circulating HGF was found in drugs targeting the MET receptor. For instance, circulating HGF was evaluated as a pharmacodynamic biomarker of MET inhibition by onartuzumab in patients with advanced cancers or with NSCLC. Administration of onartuzumab resulted in a rapid and sustained rise in HGF. It was hypothesized that onartuzumab displaced HGF binding to the MET receptor, resulting in increased circulating HGF levels. The elevation in HGF was independent of dose, drug exposure, dose duration, or tumor type and was only restricted to patients who received onartuzumab rather than those who received placebo. It was found that neither higher baseline nor elevation of HGF levels upon treatment was associated with baseline tumor burden, number of liver metastases, tumor response, or stage of the tumor at diagnosis. Evaluation of the relationship between changes in HGF levels and PFS and/or OS in MET-positive patients was inconclusive [106]. In contrast, small-molecule inhibitors targeting the MET receptor, such as tivantinib, showed no obvious changes in HGF in phase 1 clinical studies [107].

In addition, HGF has been evaluated as a potential biomarker in clinical studies for ficlatuzumab and rilotumumab, two mAbs targeting the HGF ligand. An elevation of total HGF (free plus bound) was observed with the treatment of ficlatuzumab or rilotumumab in clinical studies [44,47,48,50]. The increase in total HGF level is likely due to formation of the drug–HGF complex, which has a longer half-life than free HGF. The elevation of HGF with both rilotumumab and ficlatuzumab was not associated with clinical outcomes.

Taken together, although high HGF levels might indicate enhanced activation of the MET signaling pathway in cancer development, these data should be cautiously interpreted due to their limited predictive value for clinical response.

#### 5.1.2. MET Protein Overexpression

The current understanding is that high MET protein expression levels in tumor tissues may be associated with poor patient survival in selected cancer types.

In a phase 2 study of onartuzumab, patients whose NSCLC tissue scored as positive for MET protein by immunohistochemistry (IHC) experienced a significant benefit from treatment with onartuzumab plus erlotinib *vs.* erlotinib alone. However, mRNA expression of MET, EGFR, amphiregulin, epiregulin, and HGF did not predict survival in patients treated with onartuzumab [58]. Furthermore, phosphorylated MET, a marker for MET pathway activation, and serum MET did not appear to be suitable biomarkers for NSCLC [108]. Similarly, in a phase 2 trial, longer survival was found in patients who received rilotumumab plus ECX and had positive MET expression in tumor tissues than patients who received ECX alone and had positive MET expression in tumor tissues [51]. However, the results could not be replicated in a confirmatory phase 3 trial.

Nevertheless, MET mRNA expression showed an association with survival in newly diagnosed patients with multiple myeloma treated with bortezomib-based induction therapy. *MET* mRNA expression was higher in CD138(+) than in CD138(−) cells (*p* = 0.0009). Low MET mRNA expression characterized patients with better response (complete response or very good partial response) compared to other patients (*p* = 0.0006). Patients with high MET mRNA expression displayed worse PFS (*p* = 0.0029) and OS (*p* = 0.0023) compared to those with low MET mRNA levels. Patients with both high MET mRNA expression and high β2-microglobulin level (>5.5 mg/L) had further worse median PFS (*p* < 0.0001) and OS (*p* < 0.0001) [109].

#### 5.1.3. *MET* Gene Amplification and Mutation

The most characterized aberrancies are *MET* gene mutations, leading in most cases to a constitutively active form of the molecule, and amplification of the *MET* gene locus with overexpression of the receptor on the cell surface. On the other hand, transcriptional deregulation with overexpression of the receptor on malignant cells is probably the most common mechanism.

Mutation of the *MET* gene represents a rare event in cancer, but it might play a causal role in the development of certain cancers or lead to enhanced activation of MET kinase activity and confer tumorigenic properties in cells. High *MET* gene copy number was more frequently identified in cancers (e.g., NSCLC). *MET* gene amplifications might be associated with constitutive activation of the MET receptor and an oncogenic addiction to the MET signaling pathway [110]. Furthermore, in newly diagnosed multiple myeloma, patients carrying four *MET* gene copies (9.8%) had a short PFS [109].

In summary, circulating HGF and MET levels do not appear to be predictive biomarkers for clinical response or disease status. MET protein expression levels in tumor tissues may be prognostic biomarkers of survival in selected cancer types. *MET* gene amplification, copy number, and mutation appear to be relatively conservative biomarkers, but they may be associated with rare events in cancer development.

### 5.2. Predictive Values of HGF/MET Biomarkers for Clinical Outcome

Evaluation of the predictive values of HGF/MET biomarkers for clinical benefit is an emerging area. Some recent examples are provided here.

#### 5.2.1. Preclinical Evidence

The *in vitro* effects of onartuzumab on cell-associated and circulating MET and circulating HGF were evaluated, and results suggested that onartuzumab stably associates with MET on the surface of cells without inducing MET internalization or shedding. There was no observed effect of onartuzumab on circulating HGF levels in xenograft tumor-bearing mice or on endogenous HGF in cynomolgus monkeys; the findings were similar to those in clinical studies. It was also observed in animals that binding of MET to onartuzumab in circulation resulted in increasing MET serum concentrations due to reduced clearance of the MET-onartuzumab complex, which was also observed in the clinical setting. These preclinical findings indicated that the circulating level of HGF and MET might not be useful biomarkers for onartuzumab [108] and that preclinical investigation could provide relevant hints for clinical evaluation.

In another example, genetically engineered mouse models were used to evaluate the role of HGF overexpression in the acceleration of HCC progression. For both liver regeneration and spontaneous HCC development, there is an inclusive requirement for MET expression, and when HGF induces autocrine activation, the tumor displays sensitivity to a small-molecule MET inhibitor. The finding indicated that HGF is a driver of hepatitis B virus (HBV)-induced HCC progression and may serve as a biomarker for MET-targeted therapy. Genomic analysis showed that an upregulated HGF signature is associated with poor prognosis in HBV-positive patients with HCC [111]. Future large clinical studies could provide a confirmation of the value of this biomarker for HCC.

#### 5.2.2. Clinical Evidence

Alterations in the MET receptor might be associated with transcriptional overexpression, gene amplification, somatic or germline mutations, and/or ligand-dependent autocrine/paracrine loops. Most clinical evidence to identify predictive or prognostic values of HGF/MET biomarkers for survival was primarily based on retrospective analysis of clinical samples. Strategies in clinical studies to identify MET-driven tumors to date have focused primarily on measuring protein levels of MET, evaluating tumors for the presence of activating mutations, or assessing tumors for the presence of *MET* gene amplifications. Assessing MET expression levels by IHC will most likely be applicable to the majority of indications; gene amplification is rare (<10% in NSCLC and gastric cancer), and activating mutations are limited to few and infrequent tumor types. IHC assays to determine HER2 status in breast and gastric cancer have been in clinical practice as companion diagnostic assays and have taught us a great deal about the benefits and challenges of such an approach [110].

Recent examples of identifying potential biomarkers that could be associated with clinical outcome in various cancers are presented in Table 3.

The information presented indicates that circulating HGF and MET levels, MET protein expression levels in tumor tissues, and *MET* gene amplification, copy number and mutation, as well as markers related to crosstalk of related pathways, are commonly evaluated biomarkers. There was no universal observation in different types of cancers, and the association between biomarkers and clinical efficacy endpoints might vary with cancer types or disease stage. Therefore, specific and in-depth biomarker evaluations are needed for each cancer development.

While the benefits of biomarker selection are clear, the path to achieving the benefits is challenging. Putative biomarkers often have to be evaluated in the pivotal trial of a novel agent, at a time when many critical operating characteristics of the biomarker are unknown. There are also a variety of ways to test any given marker. Even if a novel agent makes it to the marketplace, the methodology by which a marker was assayed in a clinical trial may not mimic that which is available in regional medical communities. This leads to challenges not only in making the correct test of the marker available but also with the potential incorrect selection of patients when alternative tests are used. In addition, many drug researchers are evaluating different testing approaches for the same marker. Should multiple tests ultimately be approved for a given target, this will lead to significant challenges in cross-referencing the test developed for one agent for use with another agent, as the ability to test for the same marker with multiple different tests is constrained by both tumor tissue availability and cost.

**Table 3:** Examples of MET biomarkers in different cancers.

Cancer Type	Biomarkers (Treatment)	Key Findings
Gastric cancer	MET polymorphism (surgery)	MET polymorphism of 161 Japanese, 101 US, and 63 Austrian patients with locoregional gastric cancer treated with surgery was examined. Patients with any G (A/G or G/G genotype) allele of MET rs40239 had significantly longer disease-free survival and OS compared with those with the AA genotype in male Japanese, but not in female Japanese as well as all patients in the US and Austrian cohorts [112].
	FGFR2, HER2 and MET, tissue from tumor body (TB), luminal surface (LS), and invasive edge (IE) (surgery)	High MET expression level was associated with worse OS after adjustment for other covariates (*p* = 0.006); HER2 (*p* = 0.004) and pERK (*p* = 0.001) expression level differed between tumor regions, with increased HER2 expression level in the LS compared with the TB and IE. The potential downstream markers pS6 and pERK were expressed across tumor regions, providing evidence that resections and biopsies would yield comparative results in clinical trials [113].
	MET overexpression in tumor tissue	MET expression level was associated with improved trends in clinical outcomes [51].
Colorectal or rectal cancer	PTEN, PI3K p110a, MET, and CAIX in tumor specimens (bevacizumab)	In patients with metastatic colorectal cancer (CRC) treated with bevacizumab, expression level of CAIX, PI3K p110a, and MET in metastases did not predict objective response (OR). PTEN loss was associated with OR but not OS. Tumor heterogeneity should be taken into consideration [114].
	HGF, MET (hepatectomy)	High HGF is produced to promote liver regeneration post-hepatectomy, which was used for the treatment of liver metastasis of CRC. HGF produced after hepatectomy may stimulate the progression of CRC cells with MET in residual liver. Results from 94 CRC patients including 24 with liver metastasis showed that MET overexpression was closely associated with CRC liver metastases (87% of 24 patients), while in liver metastatic lesions, the MET expression level was reduced in comparison to primary lesions [115].
	Gene expression profiles (chemo-radiotherapy)	To differentiate gene expression profiles based on tumor regression grading (TRG) in residual cancer cells after operative chemoradiotherapy (CRT), total RNA was obtained from 52 patients with locally advanced rectal cancer to examine the expression levels of 20 genes. Expressions of *LGR5*, *PDRG1*, *GLUT*, *MK167*, and*BAX* genes were significantly associated with clinical outcome in the grading systems, while other tested gene expressions, including *HGF* and *MET*, did not show any associations, indicating that TRG may reflect features of proliferation, stemness potency, and resistance to hypoxia of residual cancer cells following preoperative CRT [116].
Papillary renal cell carcinoma (PRCC)	MET pathway activation markers (foretinib)	PRCC patients were stratified by MET pathway activation (germline or somatic *MET* mutation, *MET* [7q31] amplification, or gain of chromosome 7). The presence of a germline *MET* mutation was highly predictive of a response (five of 10 *vs.* five of 57 patients with and without germline MET mutations, respectively) [117].
Glioblastoma	HGF, *EGFRvIII* amplification (preclinical study with SGX523 and erlotinib)	HGF autocrine expression correlated with high phospho-MET levels in HGF autocrine cell lines, and these lines showed high sensitivity to MET inhibition *in vivo*. An HGF paracrine environment may enhance glioblastoma growth *in vivo* but may not indicate sensitivity to MET inhibition. *EGFRvIII* amplification predicted sensitivity to EGFR inhibition, but in the same tumor, increased copies of *MET* from gains of chromosome 7 did not increase MET activity or predict sensitivity to MET inhibitors. Thus, HGF autocrine glioblastoma bears an activated MET signaling pathway that may predict sensitivity to MET inhibitors. Serum HGF levels may be a biomarker for the presence of autocrine tumors and their responsiveness to MET therapeutics [118].
	HGF/MET (*in vitro* cell line assays)	HGF and MET are expressed *in vitro* in glioblastoma multiforme cell lines as well as in normal human astrocyte (NHA) cells. HGF stimulates tyrosine phosphorylation of MET in both glioma cell lines and NHA cells, but only the glioma cell lines proliferate and become motile and invasive in response to HGF, whereas the NHA cells are nonresponsive, implicating autocrine/paracrine MET/HGF signaling in glioma tumorigenesis; HGF signaling through MET is negatively regulated in NHA cells [119].
Breast cancer (BC)	MET tissue expression and serum level of HGF in female breast ductal carcinoma (surgery)	Significant increase in serum HGF levels was found in patients compared with healthy subjects. Serum level of HGF is an independent prognostic indicator of breast cancer. Fibrocystic disease of the breast showed weak HGFR expression, while in normal tissue, HGFR was scanty; breast invasive ductal carcinoma showed homogenous strong reaction to HGFR. Preoperative high serum HGF levels and malignancy occur together. MET overexpression in tumors was a poor prognostic factor for OS [120].
	Serum HGF level in patients and healthy subjects (surgery)	Higher serum soluble HGF was found in patients with invasive breast cancer compared with healthy subjects (*p* < 0.001). Multivariate analysis suggested that advanced tumor-node-metastasis (TNM) staging was an independent factor regarding the high level of HGF (*p* < 0.001). Preoperative serum HGF levels might reflect the severity of invasive breast cancer [121].
	MET, *p*-MET (any available treatments for BC)	High protein levels of MET and p-MET were found in 257 patients and correlated with poor prognosis for PFS and OS in HER2-positive breast cancers and associated with a significantly higher risk of recurrence and death (*p* < 0.05) [122].
Breast cancer (BC)	*MET* or *HGF* gene copy number (trastuzumab based treatment)	High gene copy number of *MET* and *HGF* was found to significantly associate with an increased risk of trastuzumab-based therapy failure (e.g., shorter time to disease progression) in HER2-positive metastatic BC. MET and HGF FISH-positive status was highly correlated (*p* < 0.001) and combination of both biomarkers did not increase predictive value of either considered separately [123].
Prostate cancer	HGF and PSA (surgery)	Combined PSA and HGF assessment increased accuracy in distinguishing patients with metastatic or localized disease [124].
Multiple myeloma	HGF (melphalan plus prednisone with or without interferon-α)	HGF was elevated at diagnosis in 43% of myeloma patients compared with healthy subjects (*p* < 0.00001). In the group with elevated HGF levels, 46% of the patients reached a plateau phase, as compared with 60% of the patients with low HGF levels (*p* = 0.005), and the median survival time was 21 and 32 months, respectively (*p* = 0.002). HGF may be a useful follow-up parameter in myeloma patients. Measurement of HGF may identify a group of patients with poor response to melphalan-prednisone treatment and short survival. HGF was a prognostic factor in patients with high levels of beta 2-microglobulin [125].
Squamous cell carcinoma of the oral tongue (SCCOT)	Expression level of MET and tumor invasion depth, lymph node metastasis (surgery)	The relationship between the expression level of MET and tumor invasion depth, lymph node metastasis, and patient survival in small (T(1-2)) SCCOT was assessed in 71 surgically treated patients. The presence of neck metastasis and >4 mm depth of tumor invasion significantly correlated with MET overexpression. The survival rates were significantly shorter in patients with MET overexpression. Constitutive activation of MET enhanced migration and invasion of tongue cancer cells *in vitro* through the expressions of matrix metalloproteinase-1, -2, and -9, and promoted tongue cancer cell growth *in vitro* and *in vivo* [126].
Endometrial cancer (EC)	HGF, MET, and basic fibroblast growth factor (bFGF) (surgery)	The prognostic significance of HGF, MET, and bFGF that contributes to angiogenesis and proliferation in numerous cancers was assessed in the tumor and stroma of EC patients (*n* = 211). Tumor bFGF was significantly associated with high-grade endometrioid and clear cell histology (*p* < 0.001), advanced stage (*p* = 0.008), positive lymph-node involvement (*p* = 0.002), poor OS (log-rank test, *p* = 0.009), and poor RFS (*p* < 0.001). HGF-positive and stromal bFGF-positive tumors had a lower risk of death compared with HGF-positive and stromal bFGF-negative tumors (hazard ratio (HR) = 0.14; 95% CI: 0.03, 0.60). HGF-positive and bFGF-positive tumors had a higher risk of recurrence compared with cases with negative expression of both markers (HR = 9.88, 95% CI: 2.63, 37.16). These data show that tumor and stromal bFGF expression level have opposite associations with survival outcomes in EC patients, which deserves confirmation in larger studies [127].

FGFR: fibroblast growth factor receptor; HGF: hepatocyte growth factor; HGFR: hepatocyte Growth Factor Receptor; EGFR: epidermal growth factor receptor; PSA: prostate-specific antigen; FISH: fluorescence *in situ* hybridization; OS: overall survival; PFS: progression-free survival; RFS: relapse-free survival; CI: confidence interval.

## 6. Challenges and Perspectives on the Development of HGF/MET Therapeutics

Although promising initial results have been reported in patients with lung, gastric, prostate, and papillary renal cancers treated with HGF/MET-targeted antagonists, optimal patient selection, diagnostic and biomarker development, and the identification and testing of optimal therapy combinations are critical for success. The wealth of basic information, analytical reagents, and model systems available concerning HGF/MET oncogenic signaling will continue to be invaluable in meeting these challenges and moving expeditiously toward more effective disease control. In this section, we discuss several challenges and perspectives on the development of HGF/MET therapeutics.

### 6.1. Select Relevant Animal Species and Preclinical Models

Selection of relevant animal species is the first issue that needs to be addressed in preclinical evaluations due to species cross-reactivity. For instance, a tissue cross-reactivity study was conducted for rilotumumab with normal tissues from mice, rats, rabbits, cynomolgus monkeys, and humans; only the patterns of tissue cross-reactivity in cynomolgus monkeys and humans were similar [128]. This created challenges in preclinical efficacy evaluation using xenograft models in mice. As a resolution, the *in vivo* antitumor activity of rilotumumab was evaluated in xenograft models in which HGF was expressed in either a paracrine or autocrine fashion, demonstrating that rilotumumab could inhibit the growth of autocrine HGF-driven glioblastomas (U-87 and U-118) and paracrine-regulated leiomyosarcoma [16,129,130]. In complement, cell-based *in vitro* functional studies were performed to determine effective concentrations for inhibiting cell proliferation and migration and for enhancing the antitumor effects of chemotherapeutic agents [130,131]. The resulting effective concentration ranges were found to align well with clinical effective doses.

### 6.2. Select Appropriate Methods to Analyze Data from Xenograft Models

It is not uncommon that a drug showing activities in a xenograft model fails to replicate the effect in humans. Xenograft models are widely used in the discovery of anticancer drug candidates. However, the limited predictive value of certain models for discerning clinical activities has been recognized as an area of improvement. It has been reported that pharmacokinetic/pharmacodynamic modeling could be used to improve predictive capability because it may provide a better understanding of the relationships between drug concentration and antitumor activities for each agent in dose-ranging xenograft or allograft experiments and because it can simulate tumor growth inhibition (TGI) in xenografts at clinically relevant regimens with predicted human pharmacokinetics. A significant correlation (*r* = 0.91, *p* = 0.0008) was observed between simulated xenograft TGI driven by human pharmacokinetics and clinical response. However, TGI observed at maximum tolerated doses in mice was not correlated with clinical response (*r* = 0.36, *p* = 0.34). Agents that lead to greater than 60% TGI in preclinical models, at clinically relevant exposures, are more likely to lead to responses in the clinic [132].

### 6.3. Identifying Signal from Noise

One of the major challenges in developing targeted therapy agents is the identification of a true signal from a noisy background. For instance, the prognostic significance of HER2 and MET protein expression level, *FGFR2* gene amplification, and downstream mediators pS6 and pERK was evaluated for their potential utility as biomarkers in patients with gastric adenocarcinoma [113]. Tissue samples from 184 patients who underwent gastric surgery were examined; *FGFR2* amplification was identified in 2% of cases and associated with worse survival (*p* = 0.005); HER2 overexpression was observed in 10% of cases and associated with increased survival (*p* = 0.041); and MET overexpression was observed in 4% of cases and associated with worse survival (*p* < 0.001). With multivariate analysis, only MET overexpression retained significance (*p* = 0.006). In contrast, pS6 and pERK levels were observed in 73% and 30% of tumors, respectively, with no association with survival. These findings implied that FGFR, HER2, and MET overexpression could be useful biomarkers only for small portion of patients. If a study enrolled unselected patients with gastric cancer, the effect of drugs targeting these specific pathways would be difficult identify due to the lack of clinical response in >90% of patients. In the development of increasingly precise therapeutics whose administration is driven by the presence of a predictive biomarker, the variability in tumor biology and prevalence of target biomarkers that exists across populations should be considered.

### 6.4. Cancer Variety with a Given Cancer Type

Variability in global treatment practices can ultimately impact the development of targeted therapeutics. These variabilities translate to complexities across a range of steps in bringing a new drug from the research setting to patient treatment centers and the commercial marketplace.

In the case of MET-targeting agents, gastric cancer is a lead indication for the class and provides a good case study of these challenges. Historically, gastric cancers are broadly classified based on histological characteristics using the Lauren and/or World Health Organization classification schemes. However, classification as one type or another does not significantly impact the clinical management of patients with gastric cancer or the development strategies of novel agents. Recently, a molecular classification has been suggested from analyses published by The Cancer Genome Atlas (TCGA) project [133]. The molecular classification divides gastric cancer into four subtypes: tumors positive for Epstein-Barr virus, microsatellite unstable (MSI) tumors, genomically stable tumors, and tumors with chromosomal instability (CIN). An integrated molecular analysis of the TCGA11 data suggests that various MET aberrations are more common with specific molecular categories. For example, MET truncation mutants seem to be more often associated with MSI tumors, whereas *MET* amplification seems to be more often associated with CIN tumors. Given that various molecular categories are likely more common in different geographic regions (e.g., EBV is endemic in certain regions), and various MET-targeting agents may be more efficacious against some MET abnormalities than others (e.g., a MET TKI may be more efficacious than an agent targeting the extracellular MET receptor domain in the case of MET autophosphorylation), these factors now have to be considered when developing a MET-targeting agent for gastric cancer. This affords the opportunity for optimizing delivery of the correct agent to the correct patient while simultaneously having significant impact on clinical trial conduct, such as what tissue analysis must be conducted, what geographies a trial should be conducted in, and what statistical assumptions should be employed.

### 6.5. Regional Differences in Cancer Treatment

Variability in local treatment practices also impacts the development of targeted agents. Again, gastric cancer illustrates this well. Gastric cancer is highly prevalent in Asian countries, such as China and Japan, and less common in Western countries. In countries such as Japan, patients with resectable disease undergo wide so-called D2 resections followed by the administration of systemic cytotoxic chemotherapy. In the West, it is more common to administer chemotherapy or chemotherapy with concurrent radiation therapy prior to surgery and to follow resection with additional chemotherapy.

In patients with metastatic disease, patients in Japan receive more lines of systemic therapy than those in the West. These differences in treatment practice seem to be responsible for the differential outcomes observed in the development of targeted therapies in Asia *vs.* non-Asian countries. For example, in the case of the ERBB2-targeting monoclonal antibody trastuzumab, a phase 3 global study comparing chemotherapy with trastuzumab *vs.* chemotherapy with placebo for patients with HER2-positive advanced gastric cancer suggested differences in the OS achieved by Asians (OS HR = 0.82) compared to non-Asians (OS HR = 0.63 in Europeans and 0.44 in Central and South Americans) [134]. A similar phase 3 study of bevacizumab suggested that patients enrolled from Asia (OS HR = 0.97) had different outcomes than those enrolled from the Americas (OS HR = 0.63) or from Europe (OS HR = 0.85) [135]. Whether or not such differences will be seen with MET-targeting agents is currently unknown and needs to be confirmed by conducting clinical trials in different regions.

The potential regional difference in biology of a disease may affect biomarker evaluation. For instance, mutations in *erbB1* (the protein product of which is EGFR) are known to be driver mutations in a subset of patients with NSCLC; these mutations are much more prevalent in Asian patients (30%–40%) than in Western patients (15%), and highly effective TKIs targeting mutant EGFR are in widespread clinical use. MET signaling is widely recognized to be an escape pathway to EGFR–TKIs, for which reason there is great interest in combining anti-MET with anti-EGFR therapies in EGFR-mutated NSCLC. Therefore, the increased prevalence in Asian patients may significantly impact where and how the trials are conducted.

## 7. Conclusions

Development of anticancer therapeutics targeting the HGF/MET pathway has become a potential strategy in pharmaceutical research in the last two decades. A number of novel therapeutic agents, either therapeutic proteins or small molecules, have been tested in patients with different tumor types in clinical studies. Promising results have been obtained in the treatment of selected cancers. Accurate identification of the sensitive patient population and treatment with the right drug or drug combination is critical for success in the clinic.
